# Effect of Caloric Restriction on Hepatic Sinusoidal System and Stellate Cells in Mice

**DOI:** 10.1155/2014/670890

**Published:** 2014-02-04

**Authors:** Jian Chen, Kara King, Jian X. Zhang

**Affiliations:** ^1^School of Human Kinetics, Laurentian University, 935 Ramsey Lake Road, Sudbury, ON, Canada P3E 2C6; ^2^Biology Department, University of North Carolina, 9201 University City Bulevard, Charlotte, NC 28223, USA

## Abstract

Aging associated changes in liver include reduced hepatic blood flow, increased number of stellate cells, and collagen deposits in perisinusoidal space. We tested the possibility of mitigating these changes with caloric restriction. Two-month-old mice were subjected to 30 percent caloric restriction for 12 months and then examined for the effect of caloric restriction on the sinusoidal network, collagen deposition, and the number of stellate cells. Using intravital fluorescence microscopy, assessments were made on sinusoidal diameter, density, volumetric flow, perfusion index, and autofluorescence of vitamin A that was primarily stored with lipid droplets in stellate cells. A significant effect was observed in the vitamin A autofluorescence of stellate cells; stellate cell associated fluorescence was diminished in terms of number and size of fluorescent spots. Caloric restriction reduced collagen deposits in liver sections and lowered the gene expression of **α**1-(I) collagen but not **α**-smooth muscle actin. No differences were detected in sinusoidal dimension measurements. Our results showed that caloric restriction was effective in ameliorating the increase in stellate cells and the mild fibrosis in old mice. However, caloric restriction had no impact on stellate cell activity level as indicated by the unaffected **α**-smooth muscle actin expression.

## 1. Introduction

Aging liver exhibits metabolic alteration, slower hepatic clearance [1], fibrosis [2], reduced blood flow, and perfusion [3]. Ultrastructure of the sinusoids also undergoes changes that are characterized with narrowing of sinusoidal diameter [17], reduced fenestration, and thickening of the endothelial wall of the capillary [4, 5]. Such changes may contribute to disorder of liver lipid metabolism [6]. Aging liver has heightened susceptibility to fibrogenic agents due to altered immune response [7]. Hepatic fibrogenic response is elevated in older patients with primary liver diseases [8, 9]. Stellate cells that store most vitamin A in the liver are primarily responsible for hepatic fibrosis [10]. There is an increase in nonactivated lipid laden stellate cells and hyperplasia in aged animals [11]. Older rats display increased autoflorescence due to accumulation of vitamin A derived metabolites in stellate cells [12].

Caloric restriction mitigates or delays many aging related alterations in gene expression that impact inflammation, cellular stress, and fibrosis in mammals [13]. However, there is very limited research into the impact of caloric restriction on sinusoidal system and stellate cells in aged animals. This study was undertaken to examine the effect of caloric restriction on fibrogenic gene expression, stellate cell, and sinusoidal dimension and perfusion.

## 2. Methods and Materials

### 2.1. Experimental Design, Caloric Restriction, and Sample Collection

The animal protocol was approved by the Institutional Animal Care and Use Committee at the University of North Carolina at Charlotte. C57BL/6J mice were placed into one of two groups at 8 weeks of age: 30% caloric restriction (*N* = 10) or ad libitum feeding (control; *N* = 10). The animals were maintained on their respective diets for 12 months. The amount of food allotted and consumed was recorded daily. The amount of food given to the individual animals in the caloric restriction group was 30% less than the recorded average food consumption by the ad libitum fed group during the week just before the start of the experiment. After the duration of the study, the microcirculation of each animal was evaluated via intravital microscopy. Subsequently, the animals were sacrificed via cardiac puncture. Liver samples were snap-frozen in liquid nitrogen immediately after collection and were stored at −80°C till use. For tissue staining, frozen tissue sections (10 *μ*m) were cut in the cryostat chamber at a temperature of −18 to −20°C and stored at −80°C until further processing. Liver sections were formalin fixed and paraffin embedded.

### 2.2. Masson's Trichrome Staining

Slides of liver sections were deparaffinized to deionized water and mordant in preheated Bouin's solution at 56°C for 15 minutes. Slides were subsequently cooled in tap water (18–26°C) in a Coplin jar. They were washed in running tap water to remove yellow color from sections and stained in working Weigert's Iron Hematoxylin Solution for 5 minutes. Next, they were washed in running tap water for 5 minutes and rinsed in deionized water. They were stained in Biebrich Scarlet-Acid Fucshin for 5 minutes, rinsed in deionized water, and placed in working Phosphotungstic/Phosphomolybdic Acid Solution for 5 minutes. Finally, slides were placed in Aniline Blue Solution, for 5 minutes and placed in 1% acetic acid for 2 minutes. The solution was discarded and the slides were rinsed, dehydrated through alcohol and xylene, and mounted. All reagents were obtained from Sigma Aldrich.

### 2.3. Intravital Microscopy

For sinusoidal epi-illumination preparation, mice fasted for 12 hours before the procedure and were allowed free access to water. They were anesthetized intraperitoneally using sodium pentobarbital (50 mg/kg body weight). After the surgery, liver lobes were examined under an Olympus IX70 inverted fluorescence microscope. For visualization of sinusoids, carboxyfluorescein diacetate succinimidyl ester (CFSE, 50 *μ*M in 0.1 mL saline) was injected intravenously. The liver surface was epi-illuminated with a 100 W mercury lamp and a filter set consisting of an excitation filter band pass 485 nm, dichroic mirror 510 nm, and barrier filter 515–565 nm. The images were processed by an ARGUS-20 image processor (Hamamatsu Photonics, Japan) at specimen-monitor ratios of ×840-2500 and projected onto a Hamamatsu C2400-08 SIT camera and viewed on a high resolution video monitor. Fields were recorded onto a VCR tape for offline analysis. Assessment of sinusoidal parameters for sinusoidal flow dynamics was done off-line from the video-recorded images using digitized frame-by-frame analysis with MetaMorph image analysis system (Universal Imaging Co., West Chester, PA, USA).

### 2.4. Quantitative Video Analysis

Sinusoid diameter and number of perfused sinusoids: for sinusoid diameter (*D*), using MetaMorph image analysis software, 5 random lines were vertically drawn across each visible sinusoid within the field. Subsequently these values were averaged to yield an average sinusoid diameter for each field. Five fields were then averaged together to yield an average diameter for each animal. Animals examined then are combined to yield a group average. The number of perfused sinusoids (*P*_*s*_) within a given microscopic field (150 *μ*m in diameter) was counted and averaged in the same way to ascertain a group average.

White blood cell velocity measurement: for white blood cell velocity (VWBC) measurements, neutrophils were labeled with carboxyfluorescein diacetate succinimidyl ester (CFSE) and tracked from the time they first appeared in the field to the time they disappeared. The velocities for each field were averaged as described for sinusoid diameter. Rolling and adhered neutrophils were excluded.

Calculation of volumetric flow and perfusion index: using the measurement of white blood cell velocity (VWBC) and sinusoidal diameter (*D*), the volumetric flow (VF) in each sinusoid was calculated based on the formula: VWBC × *π*(*D*/2)^2^. The volumetric flow was then used to generate the perfusion index by multiplying it with the number of perfused sinusoids in each group: *P*_*s*_ × VF. Intravital microscopy measurements were derived and measured as previously described [12].

Retinyl ester (vitamin A) fluorescence measurement: vitamin A within hepatic stellate cells was detected using 366 nm excitation and 450 nm emission band pass filters [14].

### 2.5. RT-PCR Analysis of *α*-Smooth Muscle Actin (*α*-SMA) and *α*1-(I) Collagen

Reverse transcriptase-polymerase chain reaction was used to determine the mRNA levels of *α*-SMA and *α*1-(I) collagen. Total RNA was isolated using TRIzol reagent (Gibco-BRL, Invitrogen, Carlsbad, CA, USA) with approximately 50 mg of whole liver tissue. Reverse transcription was performed using 1 *μ*g/*μ*L of RNA and SuperScript reverse transcriptase II. For RT-PCR, a master mix was created containing 5 *μ*L of 10x PCR buffer without MgCl_2_, 5 *μ*L 25 mM MgCl_2_, 0.5 *μ*L of 20 mM dNTP, 0.5 *μ*L of forward primer, 0.5 *μ*L of reverse primer, and 0.5 *μ*L of Taq polymerase per reaction. The sense and antisense primer sequences were for mouse *α*-SMA, SMA 5′-GCTGGACTCTGGAGATGGCCGTGAC, and 5′-CCCGAGAGGACGTTGTTAGCATAG; for mouse *α*1-(I) collagen 5′-AAACCCGAGGTATGCTTGATCTGTA, and 5′-GTCCCTCGACTCCTACATCTTCTGA, for mouse glyceraldehyde-3-phosphate dehydrogenase, GAPH 5′-CCATCACCATCTTCCAGGAGCGAG, and 5′-CACAGTCTTCTGGGTGGCAGTGAT, respectively. Hot start RT-PCR was used. cDNA was heated for 3 minutes at 75°C. The master mix was added and the subsequent reaction took place in a thermocycler. Denaturation temperature: 94°C, annealing temperature: 55°C, and extension temperature: 72°C. The housekeeping gene, glyceraldehyde-3-phosphate dehydrogenase (GADPH) was amplified to serve as an internal control for sample loading amount. The PCR products were separated by electrophoresis on a 2% agarose gel and visualized by ethidium bromide staining. All reagents were obtained from Sigma Aldrich and Fisher Scientific (Hampton, NH, USA).

### 2.6. Statistical Analysis

Data are presented as mean ± SEM. Statistical analysis was performed using either a 1-way analysis of variance (1-way ANOVA) or a 2-Way ANOVA for the caloric restriction. Statistical significance was set at *P* < 0.05. The Tukey post hoc test was used to evaluate all pairwise comparisons. Statistical analysis was performed using SigmaStat (San Jose, CA, USA).

## 3. Results

### 3.1. Caloric Restriction Reduces Hepatic Collagen Deposition and Stellate Cell Population

The visualization of in vivo autofluorescence of intracellularly stored vitamin A provided an indirect way of assessing stellate cell population [12].

**Figure 1:**
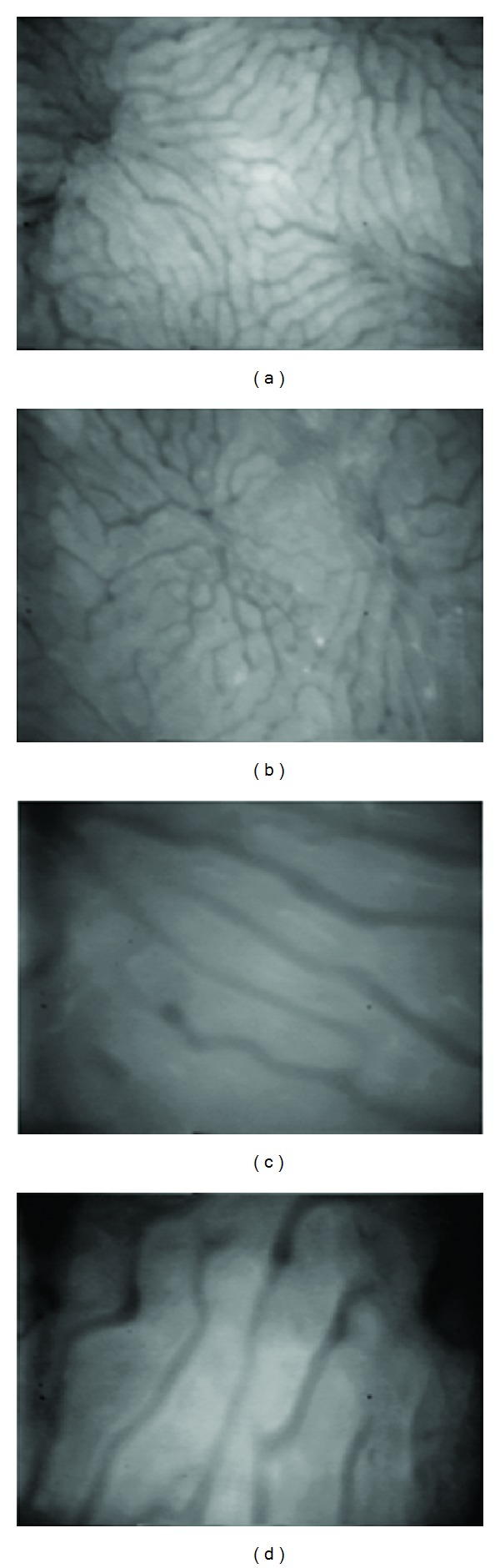
Representative intravital fluorescence microscopic images of the retinyl ester auto-fluorescence ((a) and (b)) and sinusoidal architecture ((c) and (d)). Liver from calorically restricted mice (a) shows diminished spots and intensity of vitamin A auto-fluorescence compared with control liver (b); the calorically restricted liver also has homogenous sinusoids (c) while the unrestricted liver contains more torturous sinusoids (d). Animal age is 14 months. The magnification is 20x ((a), (b)) or 100x ((c), (d)).

**Figure 2:**
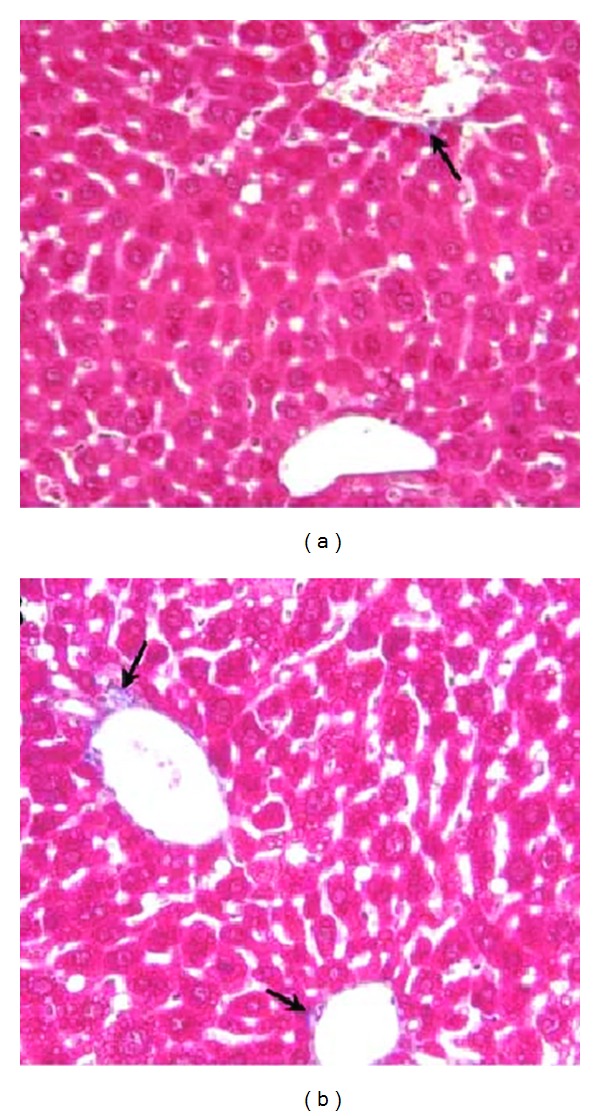
Representative images of Masson's Trichrome Stain. Collagen deposits (→) are examined on liver sections at 60x magnifications. Calorically restricted liver (a) exhibits less collagen deposits than liver of ad libitum fed animal (b); animal age is 14 months.

Figure 1 shows representative intravital fluorescence microscopic images of livers from control mice and calorically restricted mice. With CR, the number and size of fluorescence spots decreased in 14-month-old mice. Masson's Trichrome stain for collagen showed less collagen deposits in the liver sections of calorically restricted mice (Figure 2). The representative images were randomly selected sections of the liver. Compared with the control mice, there was less collagen staining in hepatic perivenular regions of the calorically restricted group (*N* = 7).

### 3.2. Effect of Caloric Restriction on *α*-SMA Induction and Fibrogenic Gene Expression

**Figure 3:**
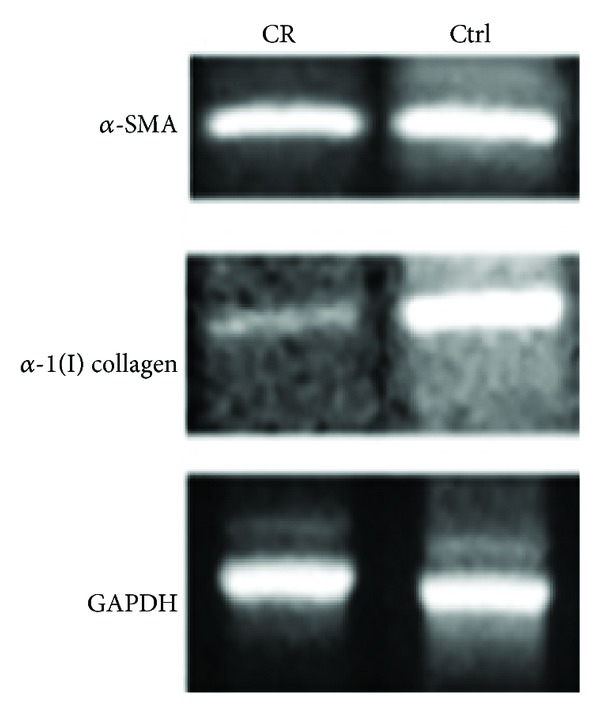
RT-PCR of profibrogenic proteins. Gene expressions of *α*-Smooth Muscle Actin (*α*-SMA, product size = 446 bp) and *α*-1(I) collagen (product size = 174 bp). The house keeping gene, glyceraldehyde-3-phosphate dehydrogenase (GAPDH, product size = 346 bp) was used to establish equal loading. The calorically restricted (CR) mice (*N* = 7) has decreased expression of *α*-1(I) collagen as compared with mice fed ad libitum (Ctrl, *N* = 7).

The induction of *α*-SMA is the single most reliable marker of stellate activation since no other cell types express *α*-SMA in liver [15]. Caloric restriction had no effect on the activation and level of stellate cells in this study. The expression of *α*-SMA was not affected by caloric restriction (Figure 3). However, the expression of *α*-1(I) collagen was lower in calorically restricted mice (Figure 3). The lowered expression of *α*-1(I) collagen provided further support for the caloric restriction effect on fibrosis, the observed decrease in collagen deposits that were identified by Masson's Trichrome stain.

### 3.3. Effect of Caloric Restriction on Sinusoids and Microcirculation

The view of intravital microscopic field revealed different appearances of sinusoidal systems between the calorically restricted animals and their unrestricted controls. The sinusoidal network of calorically restricted liver is more homogenous and less tortuous while control animals have more nonperfused areas (Figure 1).

**Figure 4:**
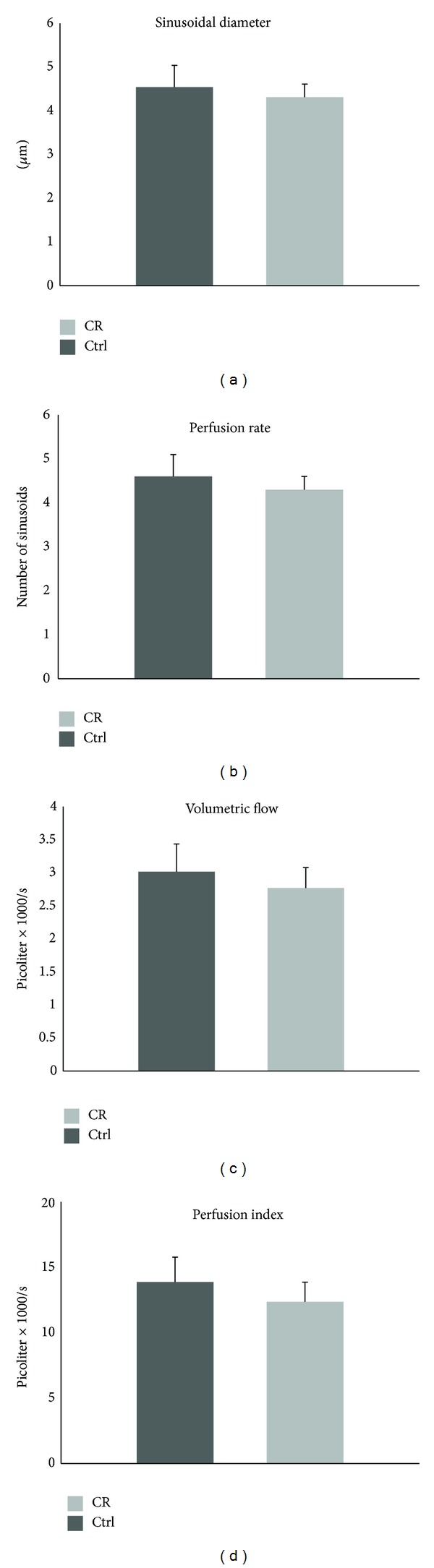
Intravital fluorescence microscopic examination of sinusoids of calorically restricted mice (CR) and control mice (Ctrl). Between the CR mice (*N* = 7) and NR mice (*N* = 7), there were so significant differences in the diameter of sinusoids (a), the perfusion rate expressed as the number of perfused sinusoids per field (b), the volumetric flows (c), and the perfusion index (d). Bars represent mean ± SEM. The mice were 14 months old.

The average sinusoidal diameters in livers of caloric restriction and control animals is 4.54 ± 0.5 *μ*m and 4.31 ± 0.3 *μ*m, respectively (Figure 4). The sinusoidal perfusion rate, expressed as the average number of perfused sinusoids per microscopic examining field, is 4.6 ± 0.4 for caloric restriction and 4.3 ± 0.3 for control groups. The volumetric flow was 3015 ± 335 picoliters per second in calorically restricted group and 2770 ± 274 picoliters per second in the control group. The calculated perfusion indexes are 13871 ± 1928 (caloric restriction) versus 12361 ± 1510 (control). Although all measurements associated with caloric restriction are higher than the control, these differences did not achieve statistical significance.

## 4. Discussion

Age associated increases in hepatic collagen deposition have been reported in several species including rat [4, 15], mouse [16, 17], and human [18, 19]. Aged liver also exhibits accumulation of stellate cells without apparent activation of these cells. Immunological staining of desmin, a stellate specific protein, revealed an increase in desmin positive cells in older mouse liver [11]. Vollmar et al. [12] reported a rise in stellate cell population in aged rats as indicated by the increased number and intensity of vitamin A autofluorescence spots. Our data demonstrated that caloric restriction ameliorated the aging related increases in hepatic stellate cell number and mild fibrosis.

The vitamin A rich stellate cells are the predominant producer of collagen fibers in liver. Once activated by injury of any etiology, they lose their storage of lipid droplets and vitamin A, transform into fibroblast-like cells, and dramatically escalate expression of fibrogenic genes [20]. Despite its well established role as a major fibrogenic cell type in liver, stellate cells may not have a significant influence on normal aging-related fibrosis. In old mice, the level of *α*-SMA expression was unchanged indicating no activation of stellate cells despite a rise of collagen content in liver [11]. In our study, even though both collagen deposits and stellate cell population were reduced by caloric restriction, *α*-SMA expression was unaffected. Therefore, the effects of normal aging and caloric restriction on fibrosis appear to be mediated by factors other than stellate cells.

Changes in collagen degradation and production of collagen by other cell types in liver may be responsible for the elevated collagen deposits. Old age was associated with increased levels of proinflammatory cytokines and inhibition of collagen degradation that can lead to fibrosis in liver [21]. Similarly, caloric restriction lowered many of the proinflammatory cytokines [13] and ameliorated age associated fibrosis [15]. Other liver cells may also contribute partly to the collagen content change in the aging liver. Liver sinusoidal endothelial cells (LSEC) play an important role in promoting inflammatory and fibrogenic milieu in liver [22]. These collagen producing cells exhibit functional changes and structural abnormality that are part of sinusoidal alteration referred as pseudocapillarization in old liver [5]. In fibrotic liver, LSEC hold several hold increases in type I collagen production [23].

Caloric restriction did not have significant impact on sinusoidal dimension and perfusion index in this study. At the end of the dietary intervention period, the mice in this study were 14 months old and were considered middle aged. It may be difficult to demonstrate a significant effect of caloric restriction on sinusoidal perfusion in the middle aged mice. Hepatic sinusoidal system of middle aged mice displayed certain functional alterations of endothelial cells and Kupffer cell phagocytic activity but had no overt changes in sinusoidal dimension and perfusion when compared with three-months-old mice [17]. In the same study [17], however, significant reduction in sinusoidal perfusion and blood flow was present in 27-month-old mice. It remains to be investigated if caloric restriction will be able to prevent or reduce alterations of sinusoidal structure and function in senescent animals.

## 5. Conclusion

The age associated increases in hepatic collagen deposition and stellate cells can be attenuated in mice by caloric restriction. However, caloric restriction had no effect on the level of stellate cell activities since the expression of *α*-SMA was unchanged in comparison with the control. Changes in LSEC collagen production and in collagen degradation may be responsible for the diminished collagen production and hepatic deposits in livers of calorically restricted animals. The caloric restriction induced reduction in stellate cells and fibrosis may lessen the fibrogenic response to pathological stimuli in old liver.
